# Synthesis of 1,3-dicarbonyl-functionalized reduced graphene oxide/MnO_2_ composites and their electrochemical properties as supercapacitors

**DOI:** 10.1039/c7ra13394d

**Published:** 2018-03-21

**Authors:** Ruiguang Xing, Ruihong Li, Xin Ge, Qiwei Zhang, Bangwen Zhang, Chaoke Bulin, He Sun, Yanan Li

**Affiliations:** School of Materials and Metallurgy, Inner Mongolia University of Science and Technology Baotou 014010 China xingrg06@imust.cn ynli2014@126.com

## Abstract

A novel 1,3-dicarbonyl-functionalized reduced graphene oxide (rDGO) was prepared by *N*-(4-aminophenyl)-3-oxobutanamide interacting with the epoxy and carboxyl groups of graphene oxide. The high-performance composite supercapacitor electrode material based on MnO_2_ nanoparticles deposited onto the rDGO sheet (DGM) was fabricated by a hydrothermal method. The morphology and microstructure of the composites were characterized by field-emission scanning electron microscopy, transmission electron microscopy, Raman microscopy and X-ray photoelectron spectroscopy. The obtained results indicated that MnO_2_ was successfully deposited on rDGO surfaces. The formed composite electrode materials exhibit excellent electrochemical properties. A specific capacitance of 267.4 F g^−1^ was obtained at a current density of 0.5 A g^−1^ in 1 mol L^−1^ H_2_SO_4_, while maintaining high cycling stability with 97.7% of its initial capacitance after 1000 cycles at a current density of 3 A g^−1^. These encouraging results are useful for potential energy storage device applications in high-performance supercapacitors.

## Abstract 2

A novel functionalized reduced graphene oxide and MnO_2_ composite was prepared as a supercapacitor electrode material.

## Graphical abstract

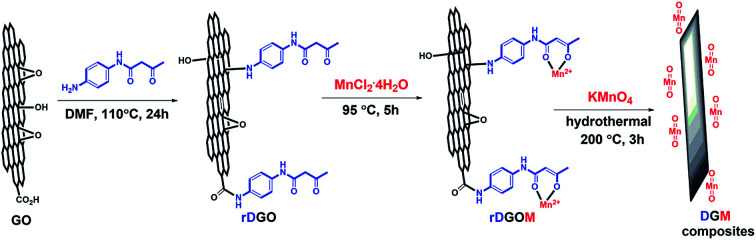

## Introduction

In recent years, along with the increasing concern about energy shortage and environment pollution, there has been an increasing demand for environmentally friendly, high performance energy-storage systems.^1,2^ Supercapacitors, one of the most promising electrochemical energy storage systems, have attracted much attention due to their higher energy density, longer cycle stability, efficiency and improved safety as compared to other batteries.^3–5^ Generally, the energy storage mechanism of supercapacitors is attributed to electric double layer capacitors (EDLCs) and pseudocapacitors.^6,7^ The typical electrode materials of EDLCs are carbon materials, which are convenient to store energy in the double layer structure formed on the surface of carbon materials. In the case of the pseudocapacitors, the most used electrode materials are conducting polymers and metal oxides, which transfer the faradaic charges between the electrolyte and electrode.^8,9^ However, each material has its unique advantages and disadvantages for application as supercapacitors.^10^ For example, carbon materials have outstanding electrical properties, long life-cycles and beneficial mechanical properties, but exhibit low specific capacitance. Transition metal oxides and conducting polymers have relatively higher capacitance and fast redox kinetics, but the relatively low mechanical stability and cycle life limit its application for supercapacitors. The challenge for the studies of composites of carbon materials, *i.e.*, combining polymers and metal oxides as materials for supercapacitors, is to effectively utilize their benefits while overcoming their disadvantages.

Graphene and its derivatives are two-dimensional carbon materials with unique mechanical and electric properties, offering a good opportunity to fabricate graphene metal oxide composites as electrode materials. Various metal oxides are used, such as tin oxide,^11^ nickel oxide,^12^ ruthenium oxide,^13^ and manganese oxide.^14–17^ Among all the transition metal oxides, manganese oxide has high specific capacitance, low cost, natural abundance, and environmental benignity and has attracted research interest as a promising material for supercapacitor applications.^18–20^

However, graphene and reduced graphene oxide (rGO) often show aggregation or restacking due to the interlayer van der Waals attractions, resulting in the deterioration of their unique properties.^21^ Thus, graphene oxide (GO), a “shining star” material, has been widely investigated as a suitable support for MnO_2_ loading.^22–24^ MnO_2_ and rGO composite for high-performance supercapacitors have been developed rapidly due to its higher electrical conductivity than GO. For example, Amir^25^*et al.* synthesized holey rGO/MnO_2_ nanosheets using the electrophoretic deposition method for high performance supercapacitors and obtained volumetric specific capacitances in the range of 182–557 F cm^−3^. Ghasemi^26^*et al.* prepared the MnO_2_/rGO nanohybrid through an electrochemically reduced process and achieved a volumetric specific capacitance of 375 F g^−1^ at a current density of 1 A g^−1^. Yang^27^*et al.* fabricated rGO and mixed-valent manganese oxide composites *via in situ* reduction by hydrazine vapour, which delivered a volumetric specific capacitance of 202 F g^−1^.

Functionalized graphene, one of the most important derivatives of graphene, is certainly key for the systematic development of graphene, which promotes pseudocapacitance. At present, few studies have been carried out on the application of functionalized graphene manganese oxide composites in supercapacitors.^28,29^

Herein, we present an approach of using 1,3-dicarbonyl-functionalized reduced graphene oxide (rDGO) and MnO_2_ composites (DGM) as electrode material for supercapacitors by a simple and feasible route. Structurally, MnO_2_ nanosheet arrays works effectively as a pseudosupercapacitor material for energy storage, while rDGO is selected as a substrate because (i) rDGO and Mn^2+^ ion have better dispersibility in solvent than graphene or reduced graphene oxide and (ii) 1,3-dicarbonyl group of rDGO and Mn^2+^ ions have strong binding force due to the coordinate bond. These features enable the subsequent *in situ* formation of MnO_2_ nanostructures on the surfaces of rDGO sheets. The electrochemical performances of the DGM electrode for supercapacitors were investigated in a three-electrode system at room temperature.

**Fig. 1:**
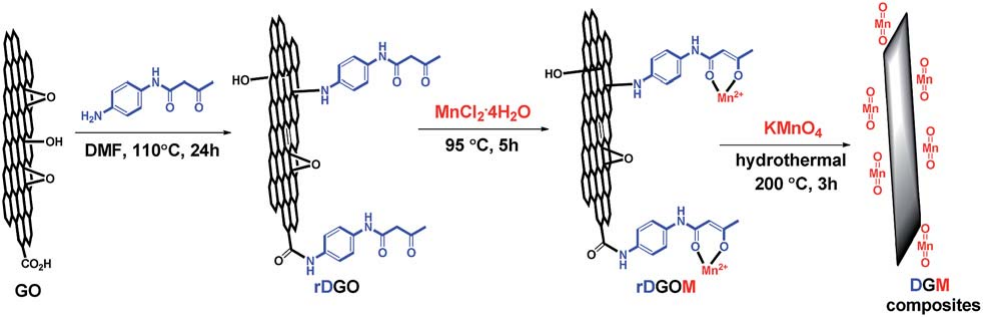
Schematic illustration for the synthesis of DGM composites.

The schematic illustration of the structure of this composite material is shown in Fig. 1. GO contains a large number of epoxy and hydroxyl groups on the basal planes and edges, and carboxyl groups on the edges. First, rDGO was synthesized by a nucleophilic reaction, which was caused by the introduced amino group of *N*-(4-aminophenyl)-3-oxobutanamide interacting with the epoxy and carboxyl groups of GO. Because the amino group of *N*-(4-aminophenyl)-3-oxobutanamide exhibits reducibility, some oxygen-containing functional groups of rDGO change to carbon–carbon double bond.^30^ Second, the rDGOM was prepared *via* the coordinate reaction of manganese chloride tetrahydrate and rDGO. The mechanism involved the ligands of 1,3-dicarbonyl group coordinating in the enolic form with manganese ion (Mn^2+^). Finally, rDGOM transformed to DGM composites for hydrothermal treatment at 200 °C for 3 h by the oxidation reduction reaction of MnCl_2_ and KMnO_4_.

## Experiments

### Materials

Flake graphite of 400 mesh was purchased from Meilikun Co. Ltd (Qingdao, China). Aqueous ammonia (28 wt%), potassium permanganate and 98 wt% H_2_SO_4_ were bought from Fengchuan Chem. Co. Ltd. (Tianjin, China). Manganese chloride tetrahydrate, *N*,*N*-dimethylformamide and *N*-(4-aminophenyl)-3-oxobutanamide were acquired from Innochem Co. Ltd (Beijing, China). All the reagents were used as received without further purification.

### Preparation of rDGO

GO was prepared by the modified Hummers' method.^31^ GO aqueous suspension (5 mg mL^−1^, 56 mL) was dispersed by ultrasonic treatment for 1 h. After the GO was entirely dispersed, *N*-(4-aminophenyl)-3-oxobutanamide (5.13 g) and *N*,*N*-dimethylformamide (DMF, 200 mL) were added to the GO suspension and was magnetically stirred at a bath temperature of 110 °C for 24 hours. Then, the mixture was repeatedly centrifuged for 15 min each at 8000 rpm with anhydrous ethanol until *N*-(4-aminophenyl)-3-oxobutanamide could not be detected in the supernatant through thin-layer chromatography (TLC) on gel F254 plates. Then, the solid underwent repeated centrifugation for 15 min at 8000 rpm with a large amount of distilled water (5 × 20 mL). The obtained rDGO colloid was divided into two parts: one part was saved in a volumetric flask (2.5 mg mL^−1^) for use in the next step; the other part was directly subjected to freeze-drying for 24 h on a vacuum freeze dryer for characterization.

### Preparation of DGM composites

For the synthesis of DGM composites, the procedure was carried out as follows: to a stirred solution of rDGO (70 mL) manganese chloride tetrahydrate (0, 100, 200, 300, 400, 500, 600 mg) was added; the reaction mixture was stirred at a bath temperature of 95 °C for 5 hours. Then, potassium permanganate (equivalent of manganese chloride tetrahydrate) was added to the above solution at room temperature. After stirring for 10 min, the mixture was transferred into a Teflon-lined stainless-steel autoclave with a capacity of 100 mL through hydrothermal treatment at 200 °C for 3 h. After the autoclave was cooled down to room temperature naturally, the precipitate was filtered under vacuum and then washed repeatedly with anhydrous ethanol and a large amount of distilled water, in sequence. Finally, the samples were directly subjected to freeze-drying for 24 h on a vacuum freeze dryer. The composite was labeled as DGM0 (0 mg)–DGM6 (600 mg). The MnO_2_ mass concentrations of DGM are 0 (DGM0), 11.6% (DGM1), 17.5% (DGM2), 24.3% (DGM3), 32.1% (DGM4), 39.3% (DGM5) and 43.8% (DGM6).

### Characterization

The functional groups were analyzed using Fourier transformation infrared spectroscopy (FTIR, Bruker IFS66V). FESEM and TEM images were obtained using a field emission scanning electron microscope (FESEM, QUANTA F250) and a transmission electron microscope (TEM, Tecnai G2F20), respectively. Chemical composition and binding energy of samples were determined using X-ray photoelectron spectroscopy (XPS) analysis (Kratos, Amicus). Raman measurements were performed using a LabRAM XploRA Raman spectrometer (HORIBA JobinYvon S. A. S).

### The electrode preparation and characterization

The electrochemical properties of the as-obtained products were investigated under a three-electrode cell configuration at room temperature. To measure the electrochemical performance of the as-synthesized DGM, DGM composites (80 wt%) were mixed with a binder (10 wt%) (polyvinylidene fluoride) and acetylene black (10 wt%). Mixing the active materials resulted in a more homogeneous slurry. The slurry was pressed on a nickel sheet to form a uniform film that was vacuum-dried at 60 °C for 12 h. A square piece of the film (1.0 cm × 1.0 cm) was cut to form the working electrode. The mass of the active material was close to 4 mg in one electrode. Before the electrochemical test, the as-prepared electrode was soaked overnight in 1 mol L^−1^ H_2_SO_4_ solution. Electrochemical characterization was carried out in a conventional three-electrode cell with 1 mol L^−1^ H_2_SO_4_ aqueous solution as the electrolyte. Platinum foil and a saturated calomel electrode (SCE) were used as the counter and reference electrodes, respectively. Cyclic voltammogram, electrochemical impedance and galvanostatic charge–discharge in different potential ranges were performed using a CHI660 electrochemical working station system (Shanghai, China) at room temperature.

### Results and discussion

**Fig. 2:**
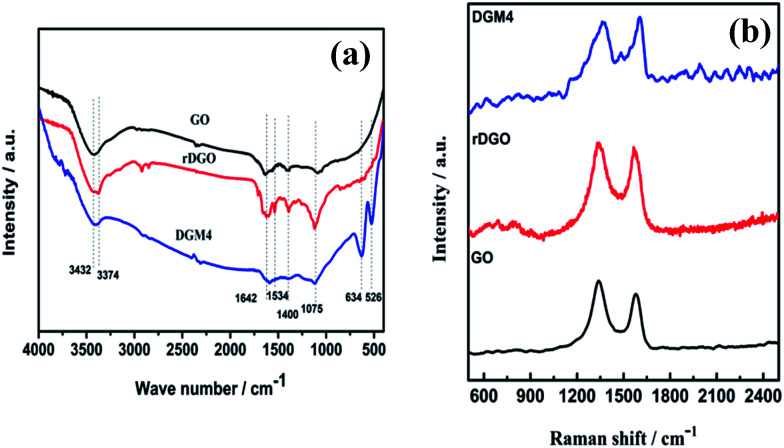
Fourier transform infrared (a) and Raman (b) spectra of GO, rDGO and DGM4.

In order to further identify the structure of MnO_2_ component in the composites, FTIR analyses of GO, rDGO and DGM4 composites were performed and the corresponding spectra are shown in Fig. 2a. In the spectrum of GO, a broad absorption band at 3432 cm^−1^ corresponds to the O–H stretching vibration, while the absorption band at 1642, 1400 and 1075 cm^−1^ can be attributed to the main characteristic groups of the C=C, C–C and C–O stretching, respectively. In contrast to the GO, the typical N–H absorption bands at 3374 and 1534 cm^−1^ of rDGO, which can be assigned to the typical stretching vibrations and bending vibrations of the amide groups, almost disappeared. This suggests that the 1,3-dicarbonyl groups on GO have been modified by *N*-(4-aminophenyl)-3-oxobutanamide. In particular, two new peaks located at 634 and 526 cm^−1^ occur in the spectra of DGM4 composites, which can be ascribed to the Mn–O and Mn–O–Mn vibrations. Raman spectra of the GO, rDGO and DGM4 are shown in Fig. 2b. Typically, the Raman peak area is quantitatively related to the concentration of the particular species. It can be clearly seen from Fig. 2b that there are two diagnostic peaks of GO centered at around 1336 and 1576 cm^−1^, corresponding to the breathing mode of *κ*-point phonons of A_1g_ symmetry and the first-order scattering of the E_2g_ phonons, respectively. The change in the *I*_G_/*I*_D_ intensity ratio suggests a change in the average size of the sp^2^ domains, which is due to a decrease in the oxygen-containing functional groups. The *I*_G_/*I*_D_ ratios of GO, rDGO and DGM4 are 0.89, 1.09 and 1.13, respectively. As compared to GO, rDGO has less oxygen-containing functional groups, which originated from the reduction of GO with the amino group of *N*-(4-aminophenyl)-3-oxobutanamide. Moreover, DGM4 composites have more carbon–carbon double bonds than rDGO, presumably due to the oxidation–reduction reaction of oxygen functional groups of rDGO and manganese ions of manganese chloride.^32^

**Fig. 3:**
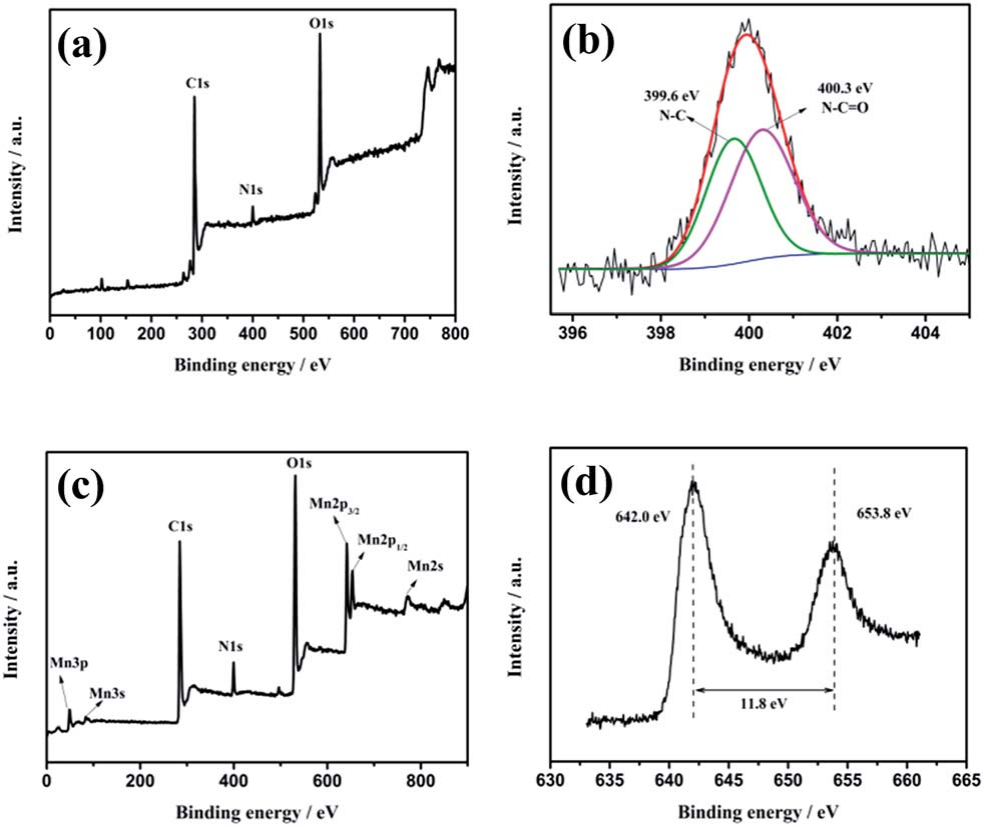
The XPS survey spectra (a) and N1s (b) core-level XPS spectra of rDGO. The XPS survey spectra (c) and Mn 2p (d) core-level XPS spectra of DGM4.

The detailed compositional analysis of rDGO and DGM4 was performed using XPS and the corresponding results are presented in Fig. 3. As shown in Fig. 3a, the XPS survey spectrum of the rDGO presents three elements, namely C, O and N. The signal of nitrogen (N 1s) emerges in XPS survey spectrum of rDGO, illustrating the attachment of *N*-(4-aminophenyl)-3-oxobutanamide on the surface and edge of GO sheets. As shown in Fig. 3b and a N 1s profile with core levels is located at around 399.6 and 400.3 eV, corresponding to the N–C and N–C=O groups, respectively, which can be clearly observed in the high resolution N 1s spectrum of the rDGO. As shown in Fig. 3c, the peaks of Mn (3p, 3s, 2p_3/2_, 2p_1/2_, 2s), O1s, C1s and N1s can be observed in the survey spectra of DGM4 composites. The peaks of Mn 2p_1/2_ and Mn 2p_3/2_ are located at 653.8 and 642.0 eV, respectively, with an energy separation of 11.8 eV (Fig. 3d), which are in good accordance with the reported data of Mn 2p_3/2_ and Mn 2p_1/2_ in MnO_2_.^33^

**Fig. 4:**
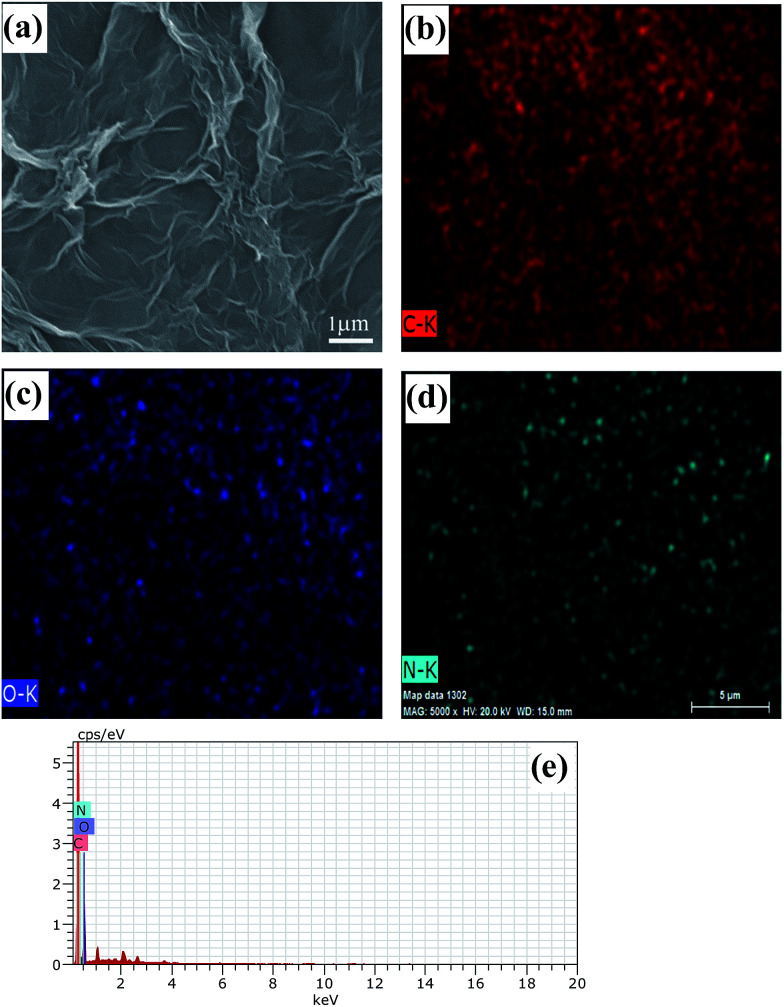
FESEM images of rDGO (a). FESEM image combined with EDS mapping in the same area and relative intensities of C (b), O (c), N (d) elements and corresponding EDS patterns (e) of rDGO.

Field emission scanning electron (FESEM) images of rDGO are shown in Fig. 4. It is clearly noted that the rDGO maintains the 2D layered flexible structure. To verify the composition of the formed compound, energy dispersive spectroscopic (EDS) mapping of the obtained material was carried out (Fig. 4b–e). These results confirm that the nitrogen atoms were distributed uniformly on the surface of the rDGO sheets. It can also be concluded that GO undergoes an efficient nucleophilic reaction with *N*-(4-aminophenyl)-3-oxobutanamide.

**Fig. 5:**
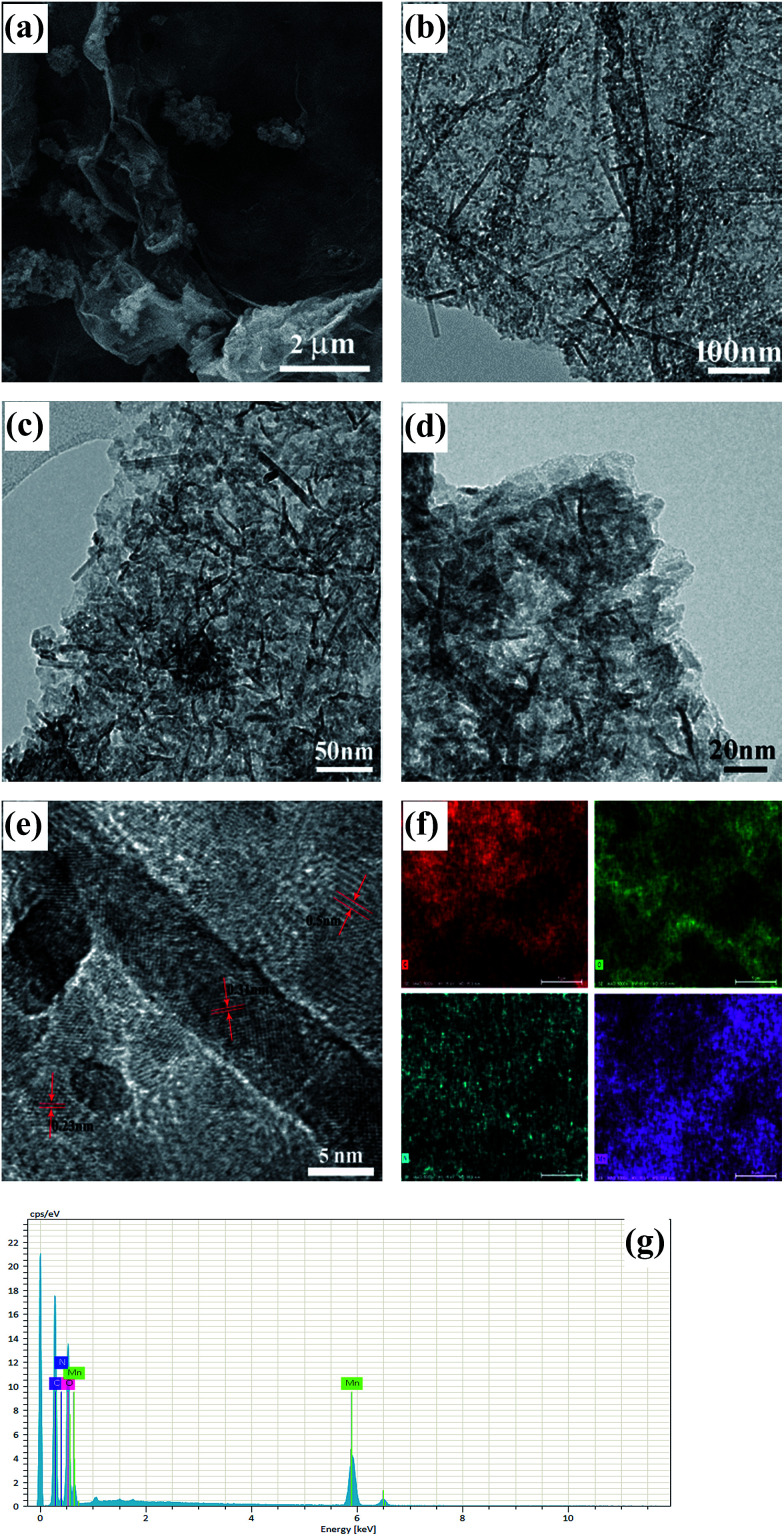
FESEM images of DGM4 (a). TEM images of DGM4 (b–e). FESEM image combined with EDS mapping in the same area and relative intensities of C (red), O (green), N (blue), Mn (purple) elements (f) and corresponding EDS patterns (g) of DGM4.

The morphology and structure of the as-prepared DGM4 composites were further studied by FESEM and TEM as shown in Fig. 5. It can be seen that flower-like MnO_2_ are decorated on the surface of rDGO and the folding and wrinkled characters of rDGO are well-preserved (Fig. 5a). However, at high magnification (Fig. 5b, c, and d), some nano-particles and a small amount of needle-like structures were observed for these flower-like MnO_2_ as shown in Fig. 5b, c and d. The distinct crystal structure of these nano-particles does not appear in the TEM image owing to its weak crystalline properties after undergoing the hydrothermal process.^5^ In addition, the needle-like crystal structure was formed due to the reaction of MnCl_2_ and KMnO_4_ before the hydrothermal process.^22^ In this case, the pseudo-capacitive character of MnO_2_ can be effectively utilized and simultaneously, rDGO also acted as an electronic conductive channel to improve the electrochemical utilization of MnO_2_. Additionally, three distinct sets of lattice spacing of *ca.* 0.23, 0.31, and 0.50 nm are marked in the nano-particles of MnO_2_, corresponding to the (211), (001), and (200) planes of α-MnO_2_, respectively (Fig. 5e). The uniform distribution of MnO_2_ can be further verified by energy-dispersive spectroscopy (EDS) elemental mapping and the corresponding EDS patterns (Fig. 5f and g, respectively). Fig. 5f and g show a FESEM image of the same area combined with EDS mapping images generated from *K*-line energy densities of C, O, N, and Mn. The results indicate that nitrogen atoms are not lost in hydrothermal condition and manganese atoms have uniform distribution on surface of rDGO, further indicating that the 1,3-dicarbonyl group of rDGO takes part in the coordination with manganese.

**Fig. 6:**
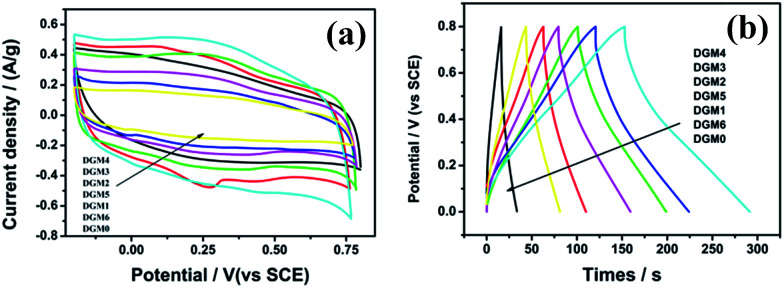
(a) Cyclic voltammogram curves of DGM with different contents of MnO_2_ at a scanning rate of 1.0 mV s^−1^, (b) galvanostatic charge–discharge curves of DGM with different contents of MnO_2_ obtained at a current density of 1.0 A g^−1^.

To explore the potential applications of the as-synthesized DGM composites, the samples were fabricated into supercapacitor electrodes and characterized with cyclic voltammogram (CV) and galvanostatic charge/discharge in 1 mol L^−1^ H_2_SO_4_ aqueous solution. The CV curves of the composites samples with different MnO_2_ contents of DGM at a scanning rate of 1 mV s^−1^ are shown in Fig. 6a. These CV curves exhibit a rectangular-like shape without distinct redox peaks, indicating ideal capacitive behaviors of the fabricated electrodes.^34^ Interestingly, with the MnO_2_ contents of DGM increasing from DGM0 to DGM6, the current densities for DGM electrodes increase first and then decrease significantly. It is well known that the specific capacitance is proportional to the area under the CV curve.^35^ These results suggest that DGM4 exhibits the largest CV area among the composites, indicating maximum capacitance due to the increase in MnO_2_ loading from DGM0-DGM4. However, greater MnO_2_-loading might lead to the agglomeration in the sample from DGM4-DGM6, resulting in low charge–transfer rate in the electrode material. This can also be confirmed from the triangular shape of the charge/discharge curves and an increase in discharge times (Fig. 6b). In addition, at a current density of 1.0 A g^−1^, the specific capacitance of DGM0, DGM1, DGM2, DGM3, DGM4, DGM5 and DGM6 are 30.4, 73.3, 146.6, 162.6, 249.4, 122.2 and 58.5 F g^−1^, respectively (calculated using data shown in Fig. 6b). Among all the composite samples, DGM4 exhibits the highest capacitance. The specific capacitance (*C*_s_) values were calculated from the charging and discharging curves (Fig. 6b) according to *C*_s_ = *I*/*m* × (d*V*/d*t*), where *I* is the constant discharge current and *m* is the mass of the active materials within the electrode. The factor d*V*/d*t* can be obtained from the slope of the discharge curve. DGM4 has the highest capacitance among the composites, matching well with the CV performances.

**Fig. 7:**
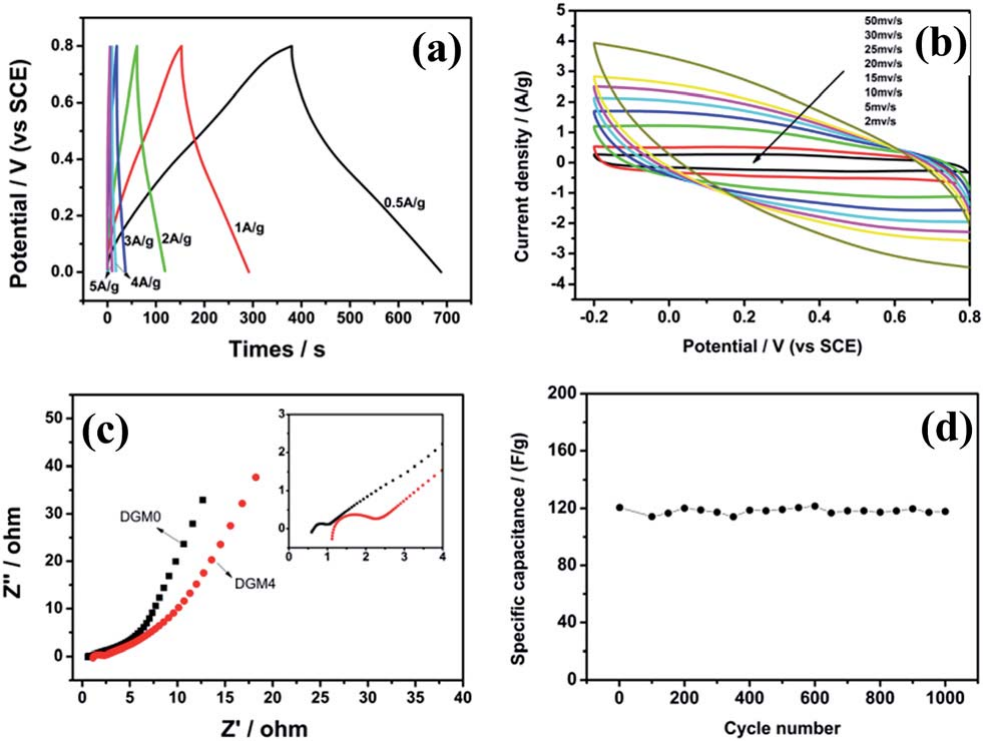
(a) Galvanostatic charge–discharge curves of DGM4 under different current densities, (b) CV curves of DGM4 at different scan rates. (c) Electrochemical impedance spectroscopies of DGM0 and DGM4 in the frequency range of 10^−2^ to 10^5^ Hz. (d) The cycle lifetime of DGM4 is at 3 A g^−1^.

On the basis of the above results, DGM4 was selected for further charge–discharge and CV performance measurements. The *C*_s_ values at 0.5, 1, 2, 3, 4 and 5 A g^−1^ are 267.4, 249.4, 212.3, 120.6, 87.65 and 60.9 F g^−1^, respectively (Fig. 7a). As expected, the *C*_s_ values decrease with an increase in current density. However, the electrode has a capacitance retention of 79% at 2 A g^−1^ based on the *C*_s_ at 0.5 A g^−1^, which demonstrates the excellent rate capability of the electrode. In addition, only 22.8% of *C*_s_ is retained when the current density increases from 0.5 to 5 A g^−1^, indicating that DGM4 composites exhibit poor electron and ionic migration performance at high current density. The typical CV curves of the DGM4 electrodes at different scan rates of 2, 5, 10, 15, 20, 25, 30 and 50 mV s^−1^ are presented in Fig. 7b. Clearly, the CV curves at all scan rates are close to a rectangular shape induced by an ideal capacitive behavior. As shown in Fig. 7c, Nyquist plots of DGM0 and DGM4 exhibit a semicircle over the high frequency range, followed by a linear part in the low frequency region with an expanded view of the high-frequency region in the inset. All plots feature the most vertical line in a low-frequency region, indicating a nearly ideal capacitive behavior. At the high frequency region, the intercept on the real impedance axis yields the electrolyte resistance (*R*_s_). The *R*_s_ values of the DGM0 and DGM4 electrodes are very low at 0.59 Ω and 1.12 Ω, respectively. Another interesting feature in the high-frequency region is that the distorted semicircle is observed for the DGM0 and DGM4 electrodes. The diameter of the semicircle gives an indication of the charge transfer resistance (*R*_ct_), which represents the electrode resistance, and is closely related to the surface area and conductivity of the electrode. The *R*_ct_ values of DGM0 and DGM4 obtained from the intersection of the Nyquist plot at the *x*-axis are 0.52 Ω and 1.24 Ω, respectively. In contrast, DGM0 exhibited a smaller *R*_ct_ value due to the good conductivity. Furthermore, the electrochemical stability of DGM4 was investigated at 3 A g^−1^ in 1 M H_2_SO_4_ aqueous solution (Fig. 7d). It was found that DGM4 electrode can retain about 97.7% (120.6 F g^−1^) of its initial capacitance after 1000 cycles, demonstrating the excellent electrochemical stability of such an electrode material.

## Conclusions

In summary, we demonstrated a novel and facile strategy to fabricate DGM4 composite from rDGO *via* the hydrothermal method. Furthermore, their structural characteristics and electrochemical properties were evaluated for potential application in supercapacitors. The uniform size and spatial distribution of MnO_2_ nanoparticles enhance the utilization of the pseudocapacitive materials, achieving the composite with a high specific capacitance, good rate capability, and long cycle life. The synthesized DGM4 is very promising for application as a high energy density supercapacitor material or other power source and the novel functionalized graphene (rDGO) will provide enlightening insights into other graphene materials.

## Conflicts of interest

There are no conflicts to declare.

## Supplementary Material
